# Enhancing Electrocatalytic Activity through Liquid‐Phase Exfoliation of NiFe Layered Double Hydroxide Intercalated with Metal Phthalocyanines in the Presence of Graphene

**DOI:** 10.1002/cphc.201900577

**Published:** 2019-08-14

**Authors:** Dulce M. Morales, Stefan Barwe, Eugeniu Vasile, Corina Andronescu, Wolfgang Schuhmann

**Affiliations:** ^1^ Analytical Chemistry – Center for Electrochemical Sciences (CES) Faculty of Chemistry and Biochemistry, Ruhr University Bochum Universitätsstr. 150 D-44780 Bochum Germany; ^2^ Department of Oxide Materials Science and Engineering University “Politehnica” of Bucharest 1–7 Gh. Polizu 011061 Bucharest Romania; ^3^ Chemical Technology III, Faculty of Chemistry and Center for Nanointegration (CENIDE) University Duisburg Essen Carl-Benz-Str. 199 D-47057 Duisburg Germany

**Keywords:** graphene, electrical conductivity, layered double hydroxide, liquid-phase exfoliation, oxygen evolution reaction

## Abstract

Earth‐abundant transition‐metal‐based catalysts are attractive for alkaline water electrolysis. However, their catalytic properties are often limited by their poor electrical conductivity. Here, we present a strategy for enhancing the electrical conductivity of NiFe layered double hydroxide (LDH) in order to further improve its properties as an electrocatalyst for the oxygen evolution reaction (OER) in alkaline media. We show that NiFe LDH containing metal tetrasulfonate phthalocyanine in the interlayers between the NiFe oxide galleries can be coupled with graphene during liquid‐phase exfoliation by taking advantage of their π‐π stacking capabilities. A substantial enhancement in the electrocatalytic activity of NiFe LDH with respect to the OER was observed. Moreover, the activity and selectivity of the catalyst materials towards the oxygen reduction reaction were investigated, demonstrating that both the metal hydroxide layer and the interlayer species contribute to the electrocatalytic performance of the composite material.

## Graphical abstract

**Stack the odds**! A strategy is presented for boosting the activity of NiFe layered double hydroxide (LDH) towards the oxygen evolution reaction. The strategy consists of enhancing its electrical conductivity by π‐π stacking graphene (G) with phthalocyanine ligands present at the LDH interlayer through liquid‐phase exfoliation. The resulting NiFe LDH/G composites exhibit remarkably higher activities than their separate components.

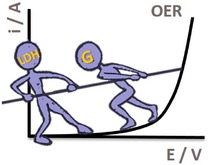

## 1 Introduction

The development of water electrolysis technologies for the clean and sustainable production of hydrogen is presently challenged by the expensiveness and low efficiencies related to the electrocatalytic oxygen evolution reaction (OER).^1^ On the one hand, its intrinsically slow reaction kinetics require large overpotentials, and on the other hand it is typically performed using noble metal‐based catalysts, which are costly and scarce.^2^ Therefore, finding alternative electrocatalysts based on widely‐available and low‐cost materials, which are able to drive the OER with comparatively low energy costs, is of great interest.

Layered double hydroxides (LDHs) are layered inorganic compounds, which possess a brucite‐like structure in which the divalent metal cations are partially replaced with trivalent cations under formation of positively charged layers. Anions intercalated between the layers compensate the charge of the positive metal hydroxide layers.^3^ LDHs containing earth‐abundant transition metals are among the best‐performing noble metal‐free OER catalysts known to date for applications in alkaline media. Tuning the catalytic properties by exchanging the anionic species within the interlayer has been demonstrated.^4^ Presently, NiFe LDH is considered as one of the state‐of‐the‐art catalysts for the OER in alkaline media.^5^ Nevertheless, as it is for most metal hydroxides, the inherently poor electrical conductivity of LDHs limits electron transfer processes during electrocatalysis, due to a high resistance impeding the electron flow across the catalyst film.^6^ Strategies to overcome the high resistance of NiFe LDH include exfoliation of the material to obtain nanosheets,^7^ and the use of conductive additives, such as graphene oxide^8^ or carbon nanotubes^9^ for enhancing the electrical conductivity of the LDHs and with this their electrocatalytic performance.

In a previous work, we proposed a method for preparing few‐layer graphene dispersions via liquid‐phase exfoliation of graphite in the presence of a metal macrocyclic complex taking advantage of their strong π‐π coupling capabilities.^10^ The role of the macrocyclic compound was twofold: to stabilize the dispersion by stacking of the macrocycle on the surface of graphene to prevent re‐agglomeration of graphene to graphite, and to serve as metal precursor for the formation of active sites. The proposed method was successful for binding metal porphyrins, metal phthalocyanines and Salen‐type structures to graphene by π‐stacking. More stable dispersions and higher graphene yields were obtained using phthalocyanine‐type ligands (Pc) containing strong electron‐withdrawing groups.^10^, ^11^ Other compounds which have been reported as effective stabilizers for graphene dispersions include surfactants, small aromatic molecules, ionic liquids, and polymers.^12^ All these compounds possess groups allowing non‐covalent interactions with graphene leading to physisorption at the surface, thus preventing restacking of the graphene sheets.^13^ Preparing graphene by means of liquid‐phase exfoliation offers several advantages compared to procedures based on e. g. the Hummers method,^14^ which are typically used for the preparation of graphene oxide and reduced graphene oxide‐based materials. For instance, the graphene quality is considerably higher when prepared via liquid‐phase exfoliation since no oxygen functional groups are introduced during synthesis, whereas graphene oxide is highly defective even after chemical reduction, thus disrupting the electronic properties of graphene. Moreover, hazardous reagents are not required, thus neither the removal of their residues, which substantially simplifies the preparation procedure.^15^

**Figure 1:**
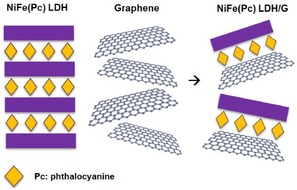
Schematic representation of liquid‐phase exfoliation of NiFe LDH containing intercalated phthalocyanine (Pc) molecules at the interlayers, in the presence of graphene to form the composite NiFe(Pc) LDH/G via non‐covalent interactions.

A material which lacks of affinity groups for low‐defect graphene could be combined with strong graphene‐coupling groups to allow it to be physisorbed at the surface of the graphene layers. An ideal candidate to prove this concept is NiFe LDH, not only because of its remarkable activity towards the OER, but also because the charge‐balancing species at the interlayer are interchangeable, thus offering the possibility of introducing graphene‐coupling groups such as Pc‐ligands at the interlayers.^16^ As depicted in Figure 1, the resulting compound (NiFe(Pc) LDH) could be coupled with graphene via π‐π stacking interactions to obtain the composite NiFe(Pc) LDH/G which exhibits an overall enhancement of the electrocatalytic performance as a result of both the intimate contact with graphene and an improved accessibility of active sites resulting from the exfoliation process.

Herein, we synthesized NiFe LDH containing either Co or Ni tetrasulfonate phthalocyanine in the interlayers, and performed liquid‐phase exfoliation to couple the LDH with graphene. We investigated the electrochemical performance of the obtained catalysts towards the OER by rotating disk electrode voltammetry demonstrating a considerable improvement of the catalytic activity upon combination of NiFe LDH with graphene.

## 2 Results and Discussion

NiFe LDH was synthesized by co‐precipitation at pH 8.5 of the respective metal nitrates (3 : 1 molar ratio) and tetrasulfonate metal phthalocyanines as precursors either containing cobalt (CoTSPc) or nickel (NiTSPc) as central metal atom. CoTSPc and NiTSPc were selected since they possess strong electron‐withdrawing groups that facilitate coupling between graphene and the phthalocyanines.^10^, ^17^ The precipitates were allowed to age for 48 h at 70 °C, subsequently collected by filtration and dried at 60 °C overnight to obtain NiFe LDH intercalated with either CoTSPc or NiTSPc. The obtained samples are denoted as NiFe(MTSPc) LDH with M=Ni or Co, according to their corresponding interlayer compound. Modification of NiFe(MTSPc) LDH samples with graphene was carried out via liquid‐phase exfoliation by sonicating the two components separately for 3 h. Subsequently, the two dispersions were mixed and sonicated for three additional hours. The resulting dispersions, which contained LDH and graphene at a mass ratio of 4 : 1, was stirred vigorously for 48 h and the products were separated and dried to obtain the composite materials modified with graphene (G), denoted as NiFe(CoTSPc) LDH/G and NiFe(NiTSPc) LDH/G.

**Figure 2:**
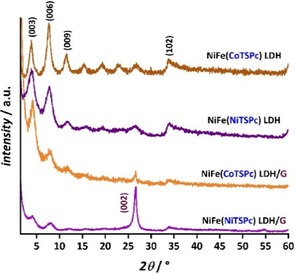
XRD patterns of NiFe(CoTSPc) LDH and NiFe(NiTSPc) LDH samples, before and after modification with graphene (G).

To assess the success of the synthesis procedure, the obtained materials were analyzed by powder X‐ray diffraction (XRD).Figure 2 shows the XRD patterns of NiFe(CoTSPc) LDH and NiFe(NiTSPc) LDH, before and after liquid‐phase exfoliation in the presence of graphene. The peaks observed at ∼4° and at ∼35° can be assigned to the LDH(Pc) reflections of the (003) and (102) planes.^18^ The presence of the harmonics of (003) are clearly distinguishable in the 2θ region from 7 to 30° for the as‐prepared NiFe(MTSPc) LDH, confirming that a well‐defined LDH structure was obtained.^18^, ^19^ The basal spacing of NiFe(CoTSPc) LDH and NiFe(NiTSPc) LDH was 22.49 and 22.84 Å, respectively, in good agreement with values reported for LDH(Pc) materials with Pc anions oriented vertically at the interlayer.^18^, ^20^ The (003) peaks positioned at 3.92 and 3.87° in the cases of NiFe(CoTSPc) LDH and NiFe(NiTSPc) LDH, respectively, were shifted to 4.22 and 4.14° after modification with graphene, respectively, leading to shorter d‐spacing values (20.93 and 21.32 Å, respectively). The shift of the (003) peak position to higher 2θ values indicates that the distance between the layers of the LDH is reduced upon modification with graphene, which is attributed to stacking between the LDH and graphene, as observed for the modification of LDH with reduced graphene oxide.^21^ In the case of the graphene‐coupled samples, a peak was observed at 2θ ≈ 26.5°, which originates from the (002) plane characteristic of graphite and multilayer graphene,^22^ with the (004) peak visible at 54.69° in the case of NiFe(NiTSPc) LDH/G. The absence of the (100) and (101) peaks in the 2θ range from 40 to 45°, in addition to the comparatively low relative intensity of the (002) and (004) peaks with respect to typical graphite XRD patterns, indicates that graphite was exfoliated to multilayer graphene.^22^, ^23^

**Figure 3:**
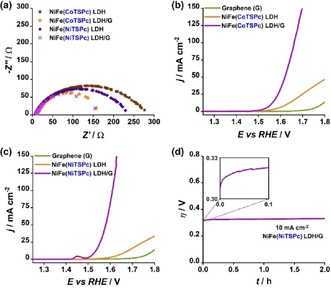
a) Nyquist plots of NiFe(MTSPc) LDH and NiFe(MTSPc) LDH/G samples obtained by galvanostatic EIS measured at 1 mA cm^−2^. Linear sweep voltammograms of NiFe(MTSPc) LDH/G samples with b) M=Co and c) M=Ni, with their respective components NiFe(MTSPc) LDH and graphene, recorded at a scan rate of 10 mV s^−1^ and an electrode rotation of 1600 rpm. d) Chronopotentiometric stability test of NiFe(NiTSPc) LDH/G conducted at a constant current density of 10 mA cm^−2^ (normalized with respect to the geometric area) and 1600 rpm rotation speed for a total duration of 2 h. The inset shows the variation of the electrode potential during the first 6 min. The measurements were carried out in aqueous 1 M KOH solution.

In the case of the composite intercalated with NiTSPc, (002) was sharper and of considerably higher relative intensity than with the CoTSPc‐containing sample, suggesting that the exfoliation process of graphite to multilayer graphene was more effective with NiFe(CoTSPc) LDH/G than with NiFe(NiTSPc) LDH/G. According to the rationale behind our material design, an increase in electric conductivity can be achieved by coupling NiFe(MTSPc) LDH with graphene, leading to an enhanced electrocatalytic activity compared to the as‐prepared samples. Electrochemical impedance spectroscopy (EIS) under OER conditions was conducted to corroborate if this was the case by comparing qualitatively the apparent change of the charge transfer resistance of the different samples. As shown in Figure 3a, the obtained Nyquist plots display larger charge transfer resistances for the as‐prepared NiFe(MTSPc) LDHs as compared to the graphene‐coupled composites, supporting our hypothesis that electron transfer processes are more favorable at NiFe(MTSPc) LDH/G samples due to a substantial difference in electric conductivity.

The activity of the NiFe(MTSPc) LDH/G composites towards the OER was evaluated by linear sweep voltammetry using rotating disk electrode (RDE) voltammetry in aqueous 1 M KOH solution as electrolyte. Figures 3b and 3c show the polarization curves obtained with NiFe(CoTSPc) LDH/G and NiFe(NiTSPc) LDH/G, respectively. Voltammograms obtained with the separate components, namely, graphene and NiFe(MTSPc) LDH, were included for comparison. The nickel oxidation peak (Ni^2+^→Ni^3+^) was clearly visible at ∼1.45 V vs RHE in the case of NiFe(NiTSPc) LDH/G (Figure 3c), whereas considerably less intense peaks were observed for the other investigated materials (Figure S1).

As clearly shown in Figures 3b and 3c, both NiFe(MTSPc) LDH/G samples outperformed NiFe(NiTSPc) LDH, NiFe(CoTSPc) LDH and graphene only, exhibiting substantially lower overpotentials for the OER. The parameter E_OER_, defined as the potential at which a catalyst attains a certain OER current density value, was used as a descriptor of the catalytic activity of the prepared samples.

The E_OER_ values obtained for all the investigated samples determined at current density values of 10 and 100 mA cm^−2^ are summarized in Table 1. NiFe(CoTSPc) LDH/G and NiFe(NiTSPc) LDH/G displayed E_OER_ values of 1.56 and 1.53 V vs RHE at 10 mA cm^−2^, respectively, which are lower by 60 and 130 mV, respectively, than those of the unsupported LDHs. The E_OER_ value of graphene at the same current density was considerably larger than all other samples (1.78 V vs RHE).

**Table 1:** OER activity descriptor of NiFe LDH samples.

Sample	E_OER_/V 10 mA cm^−2^	E_OER_/V 100 mA cm^−2^
Graphene (G)	1.78	x
NiFe(NiTSPc) LDH	1.66	x
NiFe(NiTSPc) LDH/G	1.53	1.61
NiFe(CoTSPc) LDH	1.62	x
NiFe(CoTSPc) LDH/G	1.56	1.66

x: The measured current densities were below 100 mA cm^−2^

In addition to lower overpotentials, substantially larger current densities were observed with NiFe(MTSPc) LDH/G in comparison to both the NiFe(MTSPc) LDH samples and graphene. NiFe(NiTSPc) LDH/G, which was the sample that displayed the lowest OER overpotentials, reached a current density of 100 mA cm^−2^ at an electrode potential lower than that which NiFe(NiTSPc) LDH required for reaching only 10 mA cm^−2^. Moreover, the as‐prepared NiFe(MTSPc) LDH samples were unable to reach a current density of 100 mA cm^−2^ within the investigated potential window. To evaluate the potential applicability of the obtained composites, their stability at OER conditions has to be considered. Among the investigated samples, NiFe(NiTSPc) LDH/G was the best‐performing catalyst in terms of overpotentials and current densities. To investigate the stability of NiFe(NiTSPc) LDH/G a chronopotentiometric screening procedure as proposed by McCrory *et al*.^24^ was employed, in which the electrode potential is monitored while a constant current density of 10 mA cm^−2^ is applied for a period of 2 h (Figure 3d). The catalyst displayed a total increase in overpotential of ∼20 mV by the end of the experiment. The most severe activity loss occurred within the first minutes of the experiment, with about 70 % of the total increase of overpotential being observed at t <10 min (Figure 3d, inset). At longer times, the catalyst exhibited only a mild increase in overpotential.

The activity loss of NiFe(NiTSPc) LDH/G could be explained by carbon corrosion during OER. However, other factors including accumulation of gas microbubbles on the surface of the electrode, as well as partial detachment of the catalyst film could also have a strong influence on the observed stability.^25^ Long‐term stability tests are therefore required to fully examine the performance of NiFe(NiTSPc) LDH/G. Despite of this, the synthesis allowed to successfully enhance the activity of NiFe(MTSPc) LDH‐type materials, with both NiFe(CoTSPc) LDH/G and NiFe(NiTSPc) LDH/G displaying substantially lower overpotentials and higher current densities. Moreover, differences between the OER activities of the two NiFe(MTSPc) LDH/G catalysts were observed, which suggests that the interlayer species, namely the metal phthalocyanines, contribute to the overall activity of these materials. To verify this hypothesis, we investigated the activity of the two composites towards the oxygen reduction reaction (ORR), considering that cobalt‐based electrocatalysts have demonstrated generally higher ORR performances than nickel‐based materials.^26^ We expected to observe a substantial difference in the ORR activities of NiFe(CoTSPc) LDH/G and NiFe(NiTSPc) LDH/G, provided that the interlayer species effectively play a role in the electrocatalytic process.

**Figure 4:**
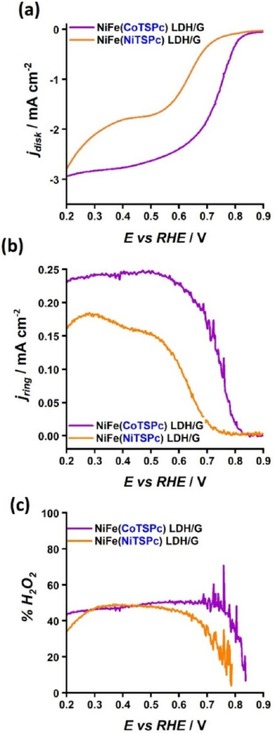
Polarization curves of NiFe LDH/G samples containing NiTSPc and CoTSPc in the interlayers, obtained by RRDE voltammetry, showing the current recorded at a) the disk and b) the ring electrode, and c) the percentage of the hydrogen peroxide yield (%H_2_O_2_). The measurements were performed at a scan rate of 10 mV s^−1^ and an electrode rotation of 1600 rpm, using O_2_‐saturated 0.1 M KOH solution as electrolyte. A constant potential of 0.4 V vs Ag/AgCl/KCl (3 M) was maintained at the ring electrode during data collection. The curves were smoothed to remove noise. Unsmoothed data is shown in Figure S2.

Investigation of the activity of the prepared composites was carried out using a rotating ring disk electrode (RRDE) setup in O_2_‐saturated aqueous 0.1 M KOH solution as electrolyte. During the experiments an oxidative potential was maintained at the ring electrode for the detection of hydrogen peroxide. Figure 4a shows linear sweep voltammograms recorded in the ORR potential region. The currents recorded concurrently at the ring electrode are shown in Figure 4b. The cobalt‐containing composite exhibited higher ORR activity than NiFe(NiTSPc) LDH/G (Figure 4a), with an overpotential difference of about 120 mV measured at a current density of −1 mA cm^−2^, indicating that the species at the interlayer are effectively participating in the catalytic processes. NiFe(CoTSPc) LDH/G displayed a diffusion‐limited current with a value close to −3 mA cm^−2^, which corresponds to a 2‐electron transfer ORR process according to the Levich equation^27^ (see Experimental Section for details). As shown in Figure 4c, maximum %H_2_O_2_ values of close to 50 % were observed for both NiFe(NiTSPc) LDH/G and NiFe(CoTSPc) LDH/G, indicating that the preferred electron‐transfer pathway was similar for the two composite materials despite of their differences in composition and activities. These results suggest that the metal hydroxide layers play a dominant role in the ORR selectivity. For a purely 2‐electron transfer pathway, %H_2_O_2_ values close to 100 % are expected. Although the observed yields of H_2_O_2_ could be an indication that both the 2‐electron and the 4‐electron transfer pathways take place concurrently, it is possible that non‐electrochemical H_2_O_2_ disproportionation occurs at the surface of the catalyst,^28^ leading to apparent lower %H_2_O_2_ values.

## 3 Conclusions

We propose a comparatively simple method for the enhancement of the electric conductivity, hence the electrocatalytic activity, of NiFe LDH by exploiting the π‐π stacking capabilities of low‐defect graphene and phthalocyanine‐type ligands present at the interlayer of the LDH. XRD analysis revealed that NiFe LDH intercalated with metal tetrasulfonate phthalocyanine (MTSPc) was successfully synthesized via co‐precipitation of metal salts and MTSPc at pH 8.5, and subsequently modified with multilayer graphene by exfoliation in liquid‐phase. Using RDE and RRDE voltammetry we demonstrated that, on the one hand, our proposed synthesis route results in a substantial improvement of the oxygen evolution activity of NiFe(MTSPc) LDH, and on the other hand, that both the metal hydroxide layer and the species in the interlayer contribute to the overall electrocatalytic performance of the composites towards both the oxygen evolution and the oxygen reduction reactions. Clearly, the proposed synthesis method is an efficient approach to enhance the activity of NiFe(MTSPc) LDH. Furthermore, the simplicity of the synthesis method makes it possible to fabricate a wide variety of LDH/G‐type catalysts with improved electrical conductivity, which could potentially find applications in different areas of electrocatalysis by varying the composition of the LDH and the species present in the interlayer, or by using graphene with different structural and catalytic properties.

## Experimental Section

### Synthesis of NiFe(MTSPc) LDH

NiFe(MTSPc) LDH was synthesized by dissolving 0.871 g (3 mmol) Ni(NO_3_)_2_ ⋅ 6H_2_O and 0.404 g (1 mmol) Fe(NO_3_)_2_ ⋅ 4H_2_O in 20 mL water in the presence of 0.372 g (2.5 mmol) triethanolamine. The mixture was added dropwise to a solution containing 0.5 mmol of either cobalt 4,4′,4′′,4′′′‐tetrasulfonated phthalocyanine (CoTSPc) or nickel 4,4′,4′′,4′′′‐tetrasulfonated phthalocyanine (NiTSPc) in 20 mL water. The pH was adjusted to 8.5 with 0.7 M NH_3_ solution using an InoLab pH Level 1 pH‐meter (WTW). The obtained mixture was stirred for 2 h at room temperature, and subsequently heated to 70 °C for 48 h. The product was washed with 250 mL water and separated by filtration using alumina membranes of 0.02 μm pore size (Whatman). After drying at 60 °C overnight, the obtained powders were ground in agate mortar in the presence of droplets of ethanol, and dried at room temperature to obtain NiFe(MTSPc) LDH with M=Co or Ni according to the corresponding precursor. The water used during the preparation of NiFe(MTSPc) LDH was boiled and purged with argon for 15 min before use.

### Synthesis of NiFe(MTSPc) LDH/G

20 mg NiFe(MTSPc) LDH samples were added to 200 mL water and sonicated for 3 h. At the same time, 5 mg high‐purity graphite (Alfa Aesar) were dispersed in 200 mL dimethylformamide and maintained under sonication for 3 h. The two dispersions were mixed and sonicated together for 3 h. During the two sonication steps, the temperature was kept below room temperature by means of an ice‐bath. The mixture was later stirred vigorously for 48 h and subsequently centrifuged at 4000 rpm for 1 h. Alumina membranes of 0.02 μm pore size (Whatman) were used for separating the product after washing with 250 mL water. NiFe(MTSPc) LDH/G samples were collected after drying the materials at 60 °C overnight. The water used during the preparation of NiFe(MTSPc) LDH/G was boiled and purged with argon for 15 min before use.

### Synthesis of Graphene

10 mg mL^−1^ high‐purity graphite were added to 400 mL DMF and sonicated for 8 h in an ice bath. The obtained graphene dispersions were centrifuged for 20 min at 4000 rpm. The supernatants were carefully collected by pipetting, and vacuum filtered using nylon membranes of 0.2 μm pore size (Whatman). The recovered powder was dried at 60 °C overnight and ground in an agate mortar.

### Structural Characterization

Powder X‐ray diffraction (XRD) patterns were recorded in the range 2θ=1‐60 ° on a Panalytical X'PERT MPD X‐ray diffractometer equipped with a Cu K‐α radiation source (λ=1.5418 Å).

### Electrochemical Characterization

All electrochemical experiments were conducted in a 3‐electrode configuration cell using an Autolab PGSTAT bipotentiostat (Metrohm) equipped with a rotator and a motor control unit. A Ag/AgCl/KCl (3 M) electrode and a platinum mesh were used as the reference and the counter electrodes, respectively. Catalyst inks were prepared by dispersing 5 mg mL^−1^ active material in a mixture of water, ethanol and Nafion (49 : 49 : 2 volume ratio) by sonication for 15 min. Rotating disk electrode (RDE) voltammetry was used for the evaluation of the OER activity and stability of the prepared samples in 1 M KOH as electrolyte. Glassy carbon RDEs (3.8 mm diameter) were polished with 0.05 μm Al_2_O_3_ paste until obtaining a mirror‐like surface, and were subsequently modified by drop‐casting 4.8 μL catalyst ink to achieve a catalyst loading of 210 μg cm^−2^. The modified RDEs were used as working electrode. Prior to the activity and stability tests, the electrodes were subjected to continuous potential cycling at 100 mV s^−1^ scan rate in the potential range between 0.1 and 0.5 V vs Ag/AgCl/KCl (3 M) until a stable response was observed, followed by electrochemical impedance spectroscopy (EIS) measured in the frequency region between 50 kHz and 10 Hz at open circuit potential with an AC perturbation of 10 mV (RMS). The uncompensated solution resistance was determined from the obtained Nyquist plots and later used for ohmic‐drop correction of the potentials. Subsequently, a linear sweep voltammogram was recorded at 10 mV s^−1^ scan rate and an electrode rotation of 1600 rpm in the potential region between 0.1 and 0.9 V vs Ag/AgCl/KCl (3 M). All samples were measured in triplicate to ensure reproducibility of the results.

The stability of the NiFe LDH was investigated chronopotentiometrically applying a constant current density of 10 mA cm^−2^ (with respect to the geometric area) for 2 h.^24^ A rotation speed of 1600 rpm was maintained throughout the experiments to prevent accumulation of gas bubbles formed during the OER. Galvanostatic EIS was used for determining the charge transfer resistance of the prepared samples. EIS were recorded in the frequency region between 1 kHz and 0.01 Hz at a current density of 1 mA cm^−2^ (with respect to the geometric area) with a perturbation current of 50 μA (RMS).

Rotating ring disk electrode (RRDE) voltammetry was used for the simultaneous investigation of the ORR activity and selectivity of the prepared samples, using aqueous 0.1 M KOH solution saturated with oxygen as electrolyte, and pre‐polished RRDEs (Autolab RRDE‐GCPt, Metrohm) with glassy carbon disk (0.1963 cm^2^) and platinum ring (0.1532 cm^2^). The disk electrode was modified with 8.31 μL catalyst ink (210 μg cm^−2^ catalyst loading) and used as working electrode. Prior to the activity and selectivity tests, the electrodes were subjected to continuous potential cycling at 100 mV s^−1^ scan rate in the potential range between 0.2 and −0.8 V vs Ag/AgCl/KCl (3 M), followed by EIS as described for OER activity measurements. Subsequently, a linear sweep voltammogram was recorded at 10 mV s^−1^ scan rate and an electrode rotation of 1600 rpm in the potential region between 0.2 and −1.0 V vs Ag/AgCl/KCl (3 M), while applying a constant potential of 0.4 V vs Ag/AgCl/KCl (3 M) at the ring electrode. The hydrogen peroxide yield (%H_2_O_2_) was calculated with the current measured at the disk (i_disk_) and at the ring (i_ring_) electrodes according to Equation (1):^29^

(1) $${\%H}_{2}O_{2}=\frac{2\;\left(i_{ring}/N\right)}{\left(i_{ring}/N\right)+i_{disk}}{}^{*}100$$

The collection efficiency factor N was determined for each catalyst film using potassium hexacyanoferrate (5 mM) dissolved in the electrolyte.^24^

All measured potentials were converted to the RHE scale and compensated for ohmic losses (iR) according to Equation (2):

(2) $$E{}_{\mathrm{RHE}}=E{}_{\mathrm{Ag}/\mathrm{AgCl}/\mathrm{KCl}}+0.207+0.059\;\mathrm{pH}-\mathrm{iR}$$

where i and R are the measured current and the uncompensated electrolyte resistance, respectively. R was obtained from the Nyquist plot resulting from EIS spectra recorded at OCP (Figure S3). The pH of 0.1 M KOH solutions was determined using a CP‐411 pH‐meter (Elmetron). For 1 M KOH solutions, the pH was estimated using Equation (3) with the average of activity of water values (γ) reported in the literature for KOH solutions.^30^ The average value used was γ=0.(3)

(3) $$\mathrm{pH}=14+\log\;[\mathrm{OH}{}^{-}]+\log\;\gamma$$

### Determination of the Diffusion‐Limited Current

The ORR diffusion‐limited current was calculated using the Levich equation [Eq. (4)], considering the Faraday constant (F), the concentration (C) and diffusion coefficient (D) of oxygen in the solution, the electrode rotation (r), and the kinematic viscosity of the electrolyte (v).^27^, ^31^

(4) $$j{}_{d}=0.21\;n\;F\;r{}^{1/2}\;D{}^{2/3}\;v{}^{-1/6}\;C$$

In the case of O_2_‐saturated KOH solutions, C, D and v have values of 1.21×10^−6^ mol cm^−3^, 1.86×10^−5^ cm^2^ s^−1^ and 1.008×10^−2^ cm^2^ s^−1^, respectively.^32^ Considering an electrode rotation of 1600 rpm, the predicted diffusion‐limited currents in the cases of 2 and 4 transferred electrons (n) are 3.09 and 6.18 mA cm^−2^, respectively.

## Conflict of interest

The authors declare no conflict of interest.

## Supporting information

As a service to our authors and readers, this journal provides supporting information supplied by the authors. Such materials are peer reviewed and may be re‐organized for online delivery, but are not copy‐edited or typeset. Technical support issues arising from supporting information (other than missing files) should be addressed to the authors.

Supplementary
