# Supplementary material for: Enhancing Electrocatalytic Activity through Liquid‐Phase Exfoliation of NiFe Layered Double Hydroxide Intercalated with Metal Phthalocyanines in the Presence of Graphene
Source: Chemphyschem. 2019 Aug 14;20(22):3030–6. doi: 10.1002/cphc.201900577 (PMC6899937; doi:10.1002/cphc.201900577)
Supplement: Supplementary file 1 — Supplementary [file CPHC-20-3030-s001.pdf]

CHEMPHYSCHEM

Supporting Information

© Copyright Wiley-VCH Verlag GmbH & Co. KGaA, 69451 Weinheim, 2019

Enhancing Electrocatalytic Activity through Liquid-Phase Exfoliation of NiFe Layered Double Hydroxide Intercalated with Metal Phthalocyanines in the Presence of Graphene

Dulce M. Morales, Stefan Barwe, Eugeniu Vasile, Corina Andronescu,* and Wolfgang Schuhmann*© 2019 The Authors. Published by Wiley-VCH Verlag GmbH & Co. KGaA. This is an open access article under the terms of the Creative Commons Attribution License, which permits use, distribution and reproduction in any medium, provided the original work is properly cited.An invited contribution to a Special Issue on Electrocatalysis

Oxidation peak of Nickel

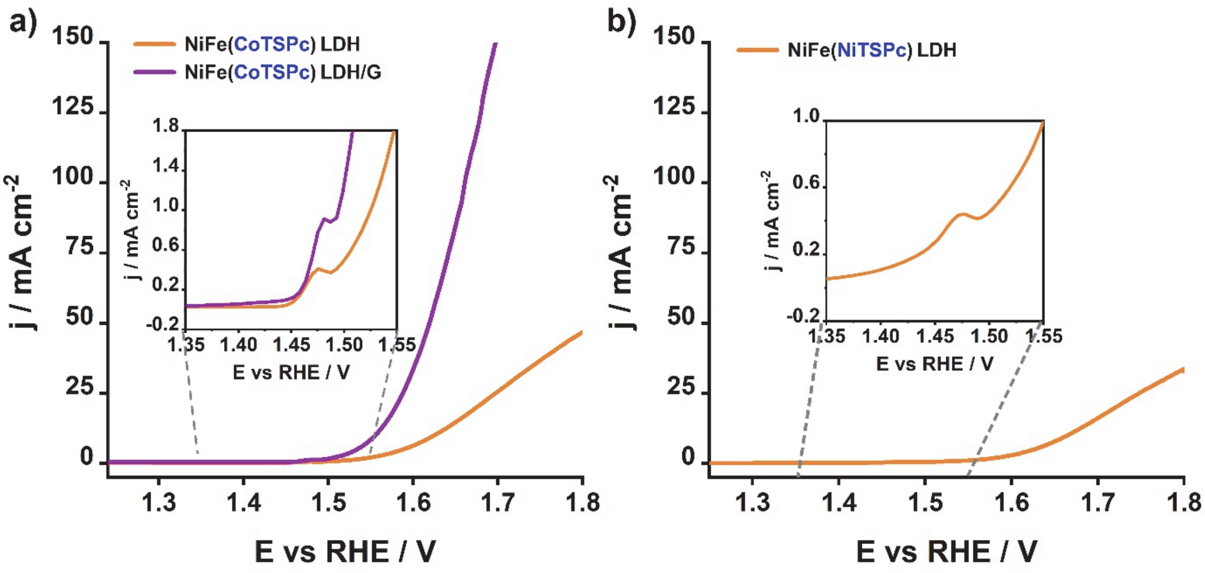

Figure S1. Linear sweep voltammograms of a) NiFe(CoTSPc) LDH and NiFe(CoTSPc) LDH/G, and b) NiFe(NiTSPc) LDH samples recorded at a scan rate of 10 mV s^{-1} and an electrode rotation of 1600 rpm. The x and y scales correspond to those from Figure 3b and 3c. The insets correspond to a zoom-in at the potential region where the oxidation peak of nickel is visible.

RRDE voltammetry - unsmoothed data

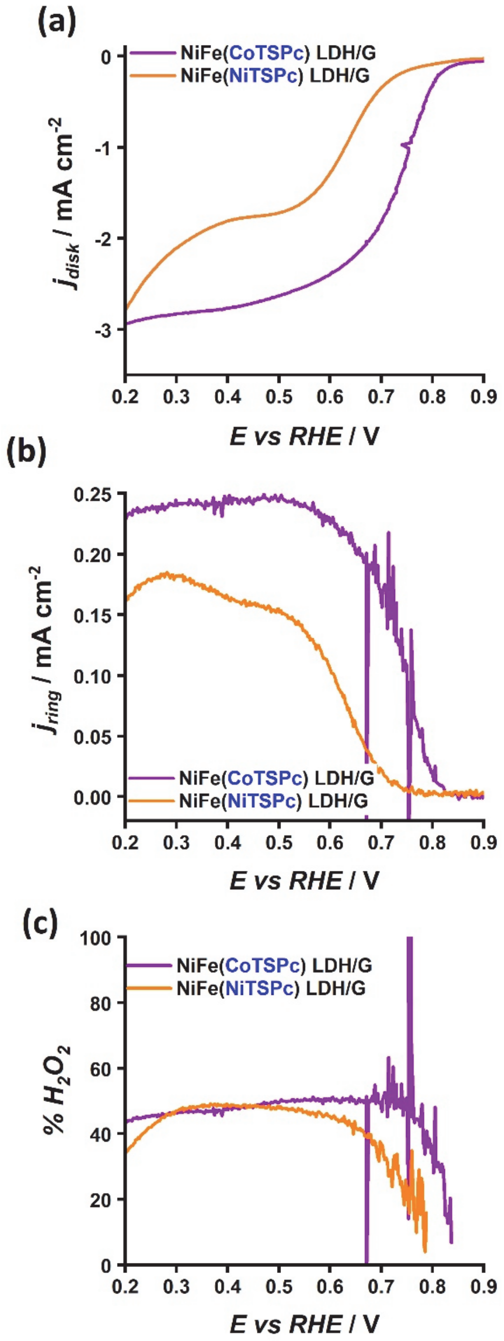

Figure S2. Polarization curves of NiFe LDH/G samples containing NiTSPc and CoTSPc in the interlayers, obtained by RRDE voltammetry, showing the current recorded at a) the disk and b) the ring electrode, and c) the percentage of the hydrogen peroxide yield (%H_2O_2). The measurements were performed at a scan rate of 10 mV s^{-1} and an electrode rotation of 1600 rpm, using O_2-saturated 0.1 M KOH solution as electrolyte. A constant potential of 0.4 V vs Ag/AgCl/KCl (3 M) was maintained at the ring electrode during data collection.

iR-drop compensation

The uncompensated resistance (R) was determined by electrochemical impedance spectroscopy (EIS). The spectra were collected in the same way for all catalysts (at open circuit potential (OCP) in the region from 50 kHz to 10 Hz with an AC perturbation of 10 mV RMS), before running other measurements. From the high frequency region of the obtained Nyquist plots, R was determined and used to correct the measured potentials as follows:

$$E_{corrected} = E_{measured} - iR$$

Additionally, galvanostatic EIS was employed for determining charge-transfer resistances of the different materials conducted at a current density of +1 mA cm^{-2} in the region from 1 kHz to 0.01 Hz with a perturbation current of 50 μA RMS. As shown in Figure S1, both the spectra collected at OCP and at +1 mA cm^{-2} show no considerable difference in the value of R, which was for all catalysts between 6 and 9 Ω.

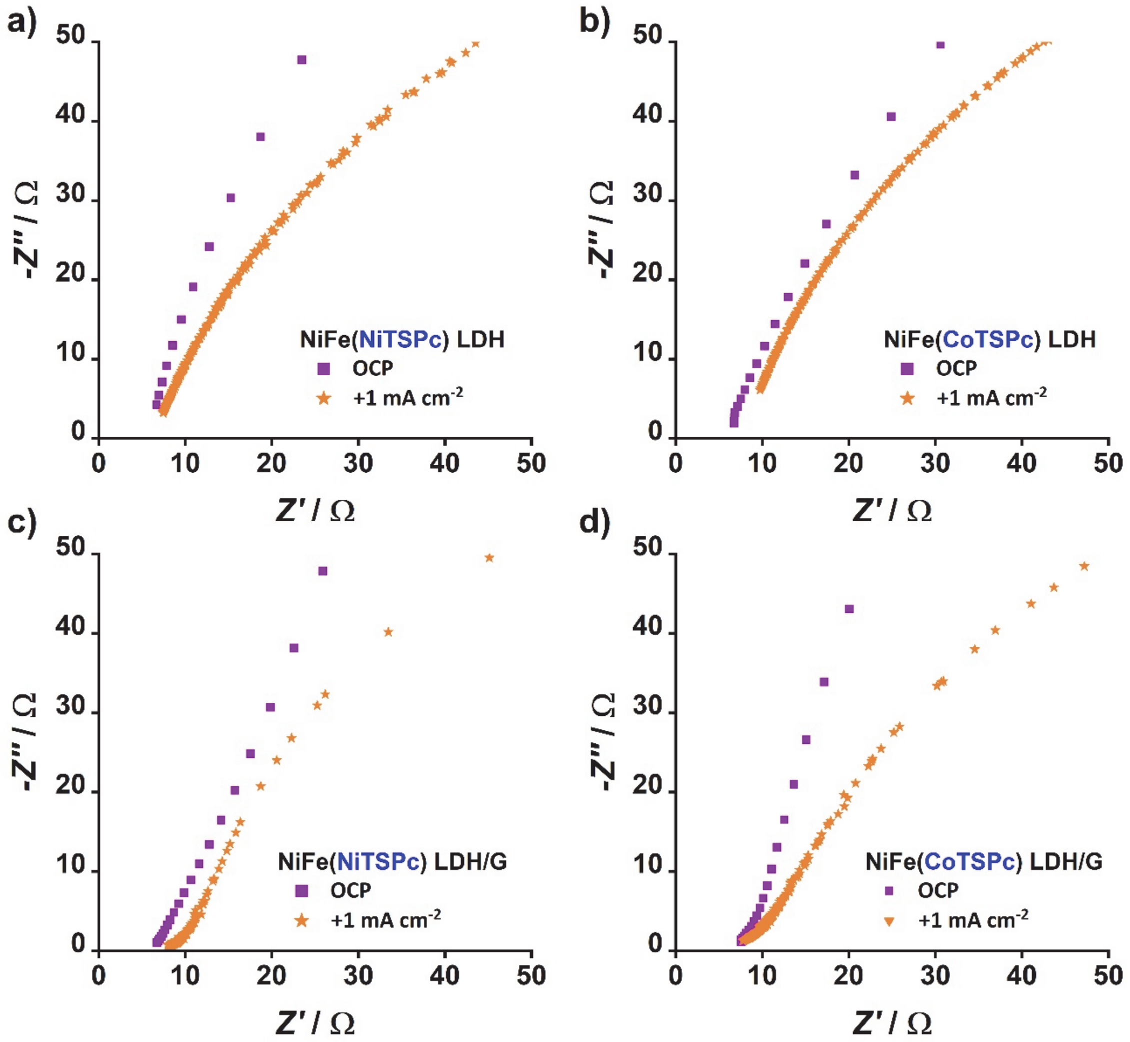

Figure S3. Nyquist plots of NiFe(MTSPc) LDH and NiFe(MTSPc) LDH/G samples obtained by potentiostatic EIS measured at OCP and galvanostatic EIS measured at 1 mA cm^{-2}.
